# Chemical/photochemical functionalization of polyethylene terephthalate fabric: effects on mechanical properties and bonding to nitrile rubber

**DOI:** 10.1038/s41598-023-41432-7

**Published:** 2023-09-04

**Authors:** Reza Ghamarpoor, Masoud Jamshidi, Mohammad Sayyadian, Mahmoud Razavizadeh

**Affiliations:** 1 Constructional Polymers and Composites Research Lab, School of Chemical, Petroleum and Gas Engineering, Iran University of Science and Technology (IUST), Tehran, Iran; 2 Department of Polymer Engineering, Faculty of Materials and Manufacturing, Malek Ashtar University of Technology, Tehran, Iran

**Keywords:** Chemistry, Engineering, Materials science

## Abstract

The aim of this work is to compare the effects of chemical and photochemical functionalization on the mechanical properties of PET fabric and its adhesion to nitrile rubber (NBR). The photochemical functionalization was performed by UV irradiation of PET fabric in the presence of glutaric acid peroxide at a temperature of 60 °C for different exposure times (i.e. 60, 90 and 120 min). The chemical functionalization (i.e. hydrolysis) of PET fabrics was performed by NaOH solution at a temperature of 60 °C for different times (i.e. 60, 120, 240 and 360 min). The tensile properties of the functionalized fibers were also evaluated. The functionalized PETs were evaluated for H-pull and T-peel adhesion to NBR. It was found that both treatment methods created functional groups on the PET surface. However, carboxylation of PET under GAP/UV irradiation generated much more OH groups on the PET surface (i.e. 4.5 times). The hydrolysis of PET in NaOH solution for more than 60 min caused a significant decrement in the tensile strength contrary to carboxylation under GAP/UV irradiation. It was also found that pullout and T-peel adhesions to NBR decreased in the case of hydrolysis of PET while they increased about 33 and 12% for GAP/UV irradiated PET, respectively.

## Introduction

Fiber-reinforced rubber composites are used in a wide variety of applications such as oil, gas, automotive and aerospace industries^1–3^. Nitrile rubber (NBR) is a fuel resistant rubber that is used in production of oil and fuel resistant O-rings, packings, sealants, hoses and tanks^4–7^. Polyethylene terephthalate (PET) is one of the most important reinforcing fabrics that is used in NBR-based composites. Since PET has a neutral and inactive nature, it has poor adhesion to the NBR compound^8^.

Many efforts have been performed to increase adhesion of different fibers/fabrics to rubbers^9–11^. Nowadays, surface modification of fibers is usually used to improve their bonding to rubbers^12,13^. Surface modification is performed through chemical^14–18^, radiation^19–23^ and thermal^24^ processes, hitherto. However, chemical methods have attracted much attention for surface modification of PET fibers in the last decades^25–27^.

It has been found that the presence of the organic groups (i.e. like coupling agents) on the PET fiber surface improves its interfacial interactions with a rubber matrix but due to its relatively passive nature, functionalization should be performed before surface modification. Hydrolysis of PET has been traditionally used for this purpose. However, it has been shown that hydrolysis process influences the PET strength properties^28,29^.

Among the various surface functionalization methods, UV-assisted treatment of PET fibers/fabrics seems a promising method according to its high performances. Liu et al.^30^ studied the surface treatment of PET fiber with succinic acid peroxide under UV light irradiation. The functionalized PETs were then reacted to methylene diphenyl di-isocyanate (MDI). It was found that fiber-rubber adhesion increased up to twice due to functionalization. Razavizadeh et al.^31,32^ functionalized PET fabric by exposing it to glutaric acid peroxide (GAP) under UV irradiation. Thereafter, the functionalized PET was grafted using MDI via a click reaction. T-peel adhesion test results showed that the surface functionalization caused more than 200% increment in PET to NBR bonding strength.

Because of the wide range of industrial applications of PET-reinforced NBR composites, the study on the surface modification of PET fiber to improve its adhesion to NBR is an interesting topic from scientific and industrial points of view. He et al.^33^ treated PET fabric with an alkaline solution and a coupling agent (i.e. KH550) with magnetic agitation. Then, it was used in the rubber matrix. The results showed that KH550 increased the adhesion of fibers to rubber by 33% compared to the sample without silane. Shao et al.^34^ were able to attach silane (i.e. KH570) to the PET fabric surface using a simple atmospheric plasma surface treatment device. They used this modified PET fabric to enhance PET-silicone rubber adhesion. The results showed that the peeling strength of the treated sample is 7.44 times that of the untreated fabric sample. Andideh et al.^35^ used bis(triethoxysilylpropyl) tetrasulfide (TESPT) with ethoxysilyl and tetrasulfide groups to strengthen short carbon fibers (CF) and styrene butadiene rubber (SBR). The results showed that the tear strength increased to 24.5% in the transverse direction of increasing the modified CF.

Studies show that the use of silane coupling agents can be a good option to increase the adhesion of PET fabric to rubber.

In this research, the effects of different treatment methods (i.e. chemical and photochemical) on the mechanical properties of PET and its adhesion to nitrile rubber were compared for the first time. For this purpose, PET fabric was hydrolyzed in NaOH solution at different times (i.e. 60, 120, 240 and 360 min). It was also surface treated in Glutaric acid peroxide (GAP) under UV irradiation as competing method for different times (i.e. 60, 90, and 120 min). The treating times were changed to optimize functionalization performances. The modified fabrics were characterized by ATR-FTIR, TGA, FE-SEM, EDS and XPS techniques. The effect of functionalization methods on the mechanical properties of PET fabrics was evaluated using the tensile test. Thereafter, the fabrics were silanized by vinyltrimethoxysilane (VTMS) to improve their bonding to NBR. Finally, bonding of PET fabrics to NBR was measured using H-pull and T-peel adhesion tests.

## Experimental

### Materials

Polyethylene terephthalate (PET) fabric prepared by HEJAB Co (Iran) was used in this research as a reinforcing textile for rubber matrix. The structural parameters of PET fabric are presented in Table 1. The fabrics were immersed in a 1 wt.% solution of non-ionic detergent in distilled water at 50 °C and then dried at 60 °C for 15 min to remove oil and pollutants.

**Table 1:** Physical properties of the used PET fabric.

Fabric type	Warp and weft density (number/10 cm)	Thickness (mm)	Linear mass density of fiber (Denier)
PET	38	0.25	50

Acrylonitrile-butadiene rubber (NBR) with acrylonitrile content of 33% and a specific density of 1.31 (g/cm^3^) (LG Company) was used as rubber in this study (see Table 2). Glutaric anhydride, hydrogen peroxide, acetone, ethanol, Sodium hydroxide (NaOH) and acetic acid were purchased from Merck Company. Vinyltrimethoxysilane (VTMS) (Evonik Company) was used as a silane coupling agent. Rubber ingredients were mixed by a laboratory two-roll mill and then mixed in a Bunbury according to ASTM D-3182 standard^36^. The prepared rubber sheets were conditioned at 25 °C for 24 h in a closed container before the determination of the optimum cure time at the temperature of 160 °C.

**Table 2:** The used nitrile rubber compound.

Ingredients	phr
NBR	100
Carbon black (N660)	65
Calcium carbonate	35
DOP oil	10
Zinc oxide	5
Stearic acid	1
Sulfur	1.2
CBS	2
TMTD	0.6

### Functionalization of PET fabrics

#### Carboxylation of PET by GAP/UV irradiation

Glutaric acid peroxide (GAP) was prepared by a reaction of glutaric anhydride with hydrogen peroxide in a water/ice bath at 0 °C^32,37^. The PET fabric was immersed in a water/acetone solution containing 5 wt% of GAP and irradiated by a UV lamp (400W, ULTRAMED400, OSRAM Co., Germany) for 60, 90, and 120 min. The water and acetone solution was placed in an ice bath to prevent temperature rising and solvent evaporation. The carboxylated fabric was washed with deionized water and dried for 15 min at 100 °C.

#### Hydroxylation of PET fabric by NaOH solution

PET fabric was immersed in NaOH solution (10 wt%) at 60 °C for 60, 120, 240 and 360 min. The hydroxylated fabric was then washed with deionized water and dried for 15 min at 100 °C^14,38^.

#### Silanization of PET fabric

Water and ethanol (solvent) were firstly combined at a volume ratio of 20:80. The pH of the mixture was adjusted to 3–4 using acetic acid and then VTMS (at 1 wt%) was added dropwise to the solution. The silane solution was mixed for 60 min to complete the hydrolysis process. The functionalized fiber/fabric was immersed in the silane solution at ambient temperature for 60 min. Thereafter, the modified fabrics were washed with deionized water to remove the solvents and unreacted silane molecules. They were then subjected to 100 °C for 30 min in an oven for condensation of silane molecules^36,39,40^.

Considering constant parameters such as time, temperature, pressure and amount of solvent, the optimal coefficient of the relationship (i.e. X number) was calculated. The most important unknown in Eq. (1) to obtain the amount of silane was the number of hydroxyl and carboxyl groups (−OH and −COOH) per gram unit of fabric (*n*_*OH*_). This parameter was calculated using TGA test data and Eq. (2), in the way that for each water molecule separated from the surface in the specified temperature range, two surface −OH groups are separated^41,42^.

1 $$ m_{VTMS} = X \times \left( {\frac{{M_{VTMS} \times m_{PET} \times n_{OH} }}{{N_{A} }}} \right). $$

2 $$ n_{OH} = \frac{{2 \times N_{A} \times W_{{{\text{H}}_{2} {\text{O}}}} }}{{18 \times W_{final} }}. $$

The value of $W_{{H_{2} O}}$ was obtained from Eq. (3) and fm the TGA test results for functionalized PET fabric (hydroxylated and carboxylated) in the temperature range of 120–350 °C. The number 18 was the molecular mass of water and its unit is grams per mole^5^.

3 $$ W_{H2O} = \left( {\Delta m_{modofied PET } - \Delta m_{pristine PET} } \right) $$

$\Delta m_{modofied PET }$ was the weight loss percentage of carboxylated or hydroxylated fabric in the temperature range of 120–350 °C. $\Delta m_{pristine PET}$ was the weight loss percentage of PET fabric without surface treatment in the range of 120–350 °C.

### Tests and characterizations

Tensile test was performed on the pristine PET fiber and functionalized PET fibers in NaOH solution and GAP/UV irradiation according to the ASTM-D2256 standard at a test speed of 10 mm/min. H-pull test of PET cords from NBR rubber was performed according to ASTM-D4776 standard at ambient temperature with a pulling speed of 120 mm/min. Adhesion of the pristine, functionalized and silanized PET fabrics to NBR were evaluated by T-peel adhesion test according to ASTM D 413 standard at ambient temperature with a separation speed of 50 mm/min.

Attenuated total reflectance infrared spectroscopy (FTIR-ATR) was carried out on the PET fabrics before and after treatments by Bruker EQUINOX 55 spectrometer. The surface of the treated and untreated fabrics was studied using a TESCAN-Mira III Field Emission-Scanning Electron Microscope (FE-SEM) equipped with energy-dispersive X-ray spectroscopy (EDX) device. Thermal Gravimetric Analysis (TGA) was performed with METTLER-TOLEDO analyzer at a heating rate of 10 °C/min under airflow from 50 to 600 °C.

## Results and discussions

### Characterization of functionalized PET

**Figure 1:**
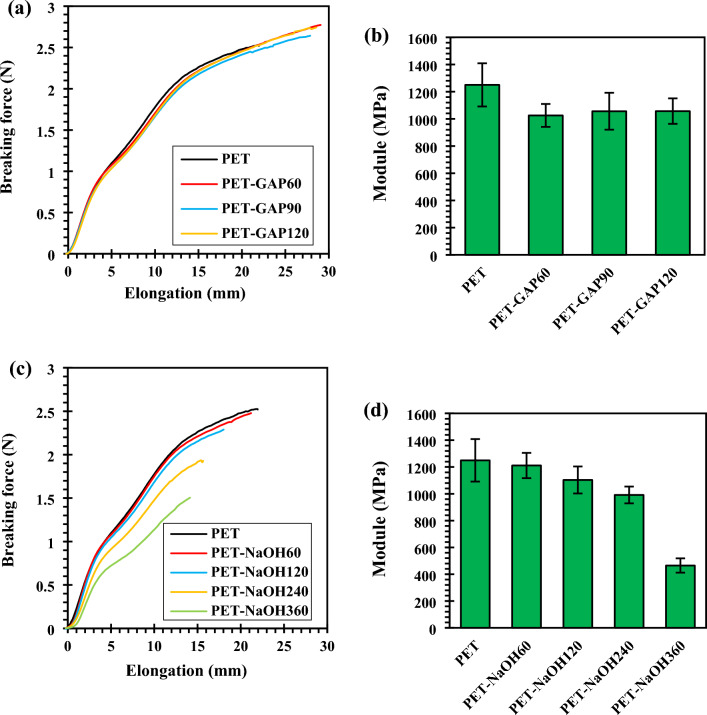
Tensile strength and modulus of the functionalized PET fabrics by; (**a**,**b**) GAP/UV irradiation and (**c**,**d**) NaOH.

The tensile properties of pristine and functionalized PET fabrics were shown in Fig. 1. Results showed that carboxylation under GAP/UV irradiation caused a slight increment in the tensile strength but decrement in the modulus of the PET fabrics (see Fig. 1a,b). This was attributed to the influence of UV waves on the relaxation of PET chains from interlocks that caused chains movement but decreased the fibers crystallinity^32^.

Figure 1c illustrates that hydroxylation by NaOH caused a decrement in the tensile strength of PET fabric. Moreover, considerable decrements in the modulus of the PET fabrics were observed at higher exposure times (see Fig. 1d). This corresponded to the internal phase hydrolysis of fibers in NaOH solution. However, hydrolysis at durations less than 120 min caused less fall in the tensile strength compared to the photo-chemically functionalized fabrics. On this basis, the samples that hydrolyzed for 60 and 120 min were selected for the next analysis and tests.

**Figure 2:**
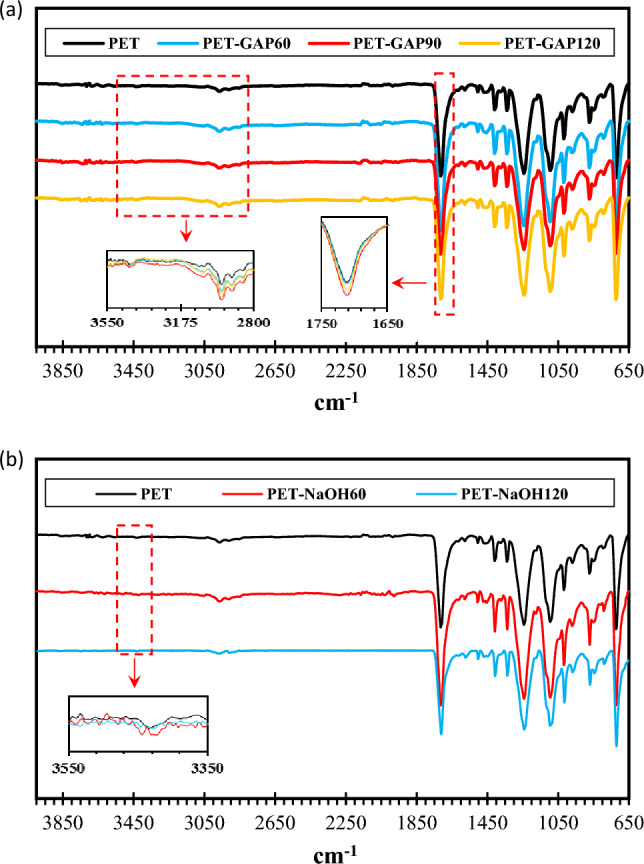
FTIR Analysis of the functionalized PET fabrics by; (**a**) GAP/UV irradiation and (**b**) NaOH.

Also, ATR-FTIR analysis of the functionalized fabrics was investigated. It was found that there are low OH groups on the pristine PET surface due to the weak peak appeared at the wavelength of 3500 cm^−1^. However, the OH content was increased by UV–assisted functionalization. The peak appeared at about 1720 cm^−1^ was attributed to carbonyl group (–C=O) in the ester groups in PET structure that increased by functionalization due to the formation of COOH groups on the surface layer (see Fig. 2a).

Figure 2b also presents the ATR-FTIR spectrums of hydroxylated PET fabrics. It is clearly seen that PET-NaOH60 sample shows intensified peaks at 3500 cm^−1^ and 1720 cm^−1^ that confirms better hydroxylation of PET surface in NaOH for 60 min compared to 120 min.

**Figure 3:**
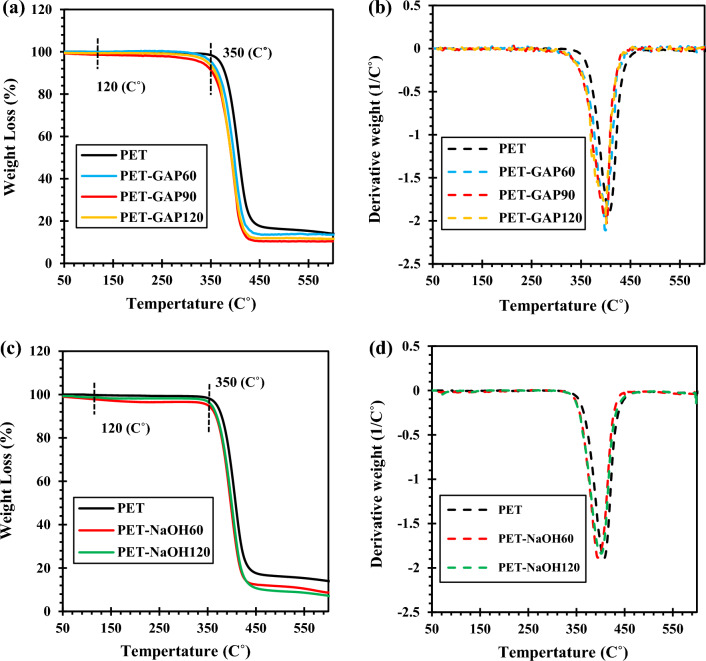
TGA and DTG analysis of the functionalized PET fabrics; (**a**,**b**) by NaOH and (**c**,**d**) by GAP/UV irradiation.

Derivative thermogravimetric (DTG) results of the functioned PET fabrics were investigated in Fig. 3. The results are listed in Table 3.

**Table 3:** TGA data for surface treated PETs**.**

Sample	Weight loss (%) (120–350 °C)	$$W_{{{\text{final}}}}$$ in 350 (C˚)	$$W_{{{\text{H}}_{2} {\text{O}}}}$$(%)	$$n_{{{\text{OH}}}}$$$$\times N_{{\text{A}}} \left( {{\text{gr}}^{ - 1} } \right)$$
PET (Pristine)	1.35	98.41	–	–
PET-GAP60	4.61	95.41	3.26	0.004
PET-GAP90	7.35	91.24	6	0.007
PET-GAP120	6.01	93.32	4.66	0.005
PET-NaOH60	2.63	95.7	1.28	0.0015
PET-NaOH120	1.63	97.38	0.25	0.0003

*NA* Avogadro number.

Figure 3a shows TGA curves of PET fabrics exposed to GAP/UV irradiation at different times. The weight loss between 50–120, 120–350 and 350–430 °C were attributed to the evaporation of humidity and low molecular weight organic matters, detachment of covalently bonded OH groups (i.e. related to –COOH groups) and PET degradation, respectively^43^.

Decreasing weight at the temperature range of 120–350 °C which is related to the detachment of covalently bonded OH groups to PET surface confirmed the successful functionalization of PET by GAP/UV irradiation at different exposure times. Besides, the PET fabric exposed to GAP/UV irradiation for 90 min showed the highest functionalization content. On this basis, this sample was selected as the best carboxylated fabric and used in silanization step. The number of OH groups per gram of PET was also calculated based on the weight loss in a temperature range of 120–350 °C the results are illustrated in Table 3.

DTG results showed that photochemical functionalization did not affect the crystallinity of the PET fabric due to negligible difference in the area under the endothermic curves (see Fig. 3b) but it decreased the degradation temperature somehow.

Figure 3c demonstrates TGA curves of hydroxylated PET fabrics. Results showed that the hydroxylation process also created hydroxyl (OH) groups on the PET surface. Moreover, the sample that was treated for 60 min showed higher OH content (see Table 3). Figure 3d represents that the degradation temperature of PET slightly decreased due to hydrolysis.

Comparing the weight loss for the chemically and photo-chemically functionalized fabrics shows that more functional groups formed on the PET surface via the later method.

**Figure 4:**
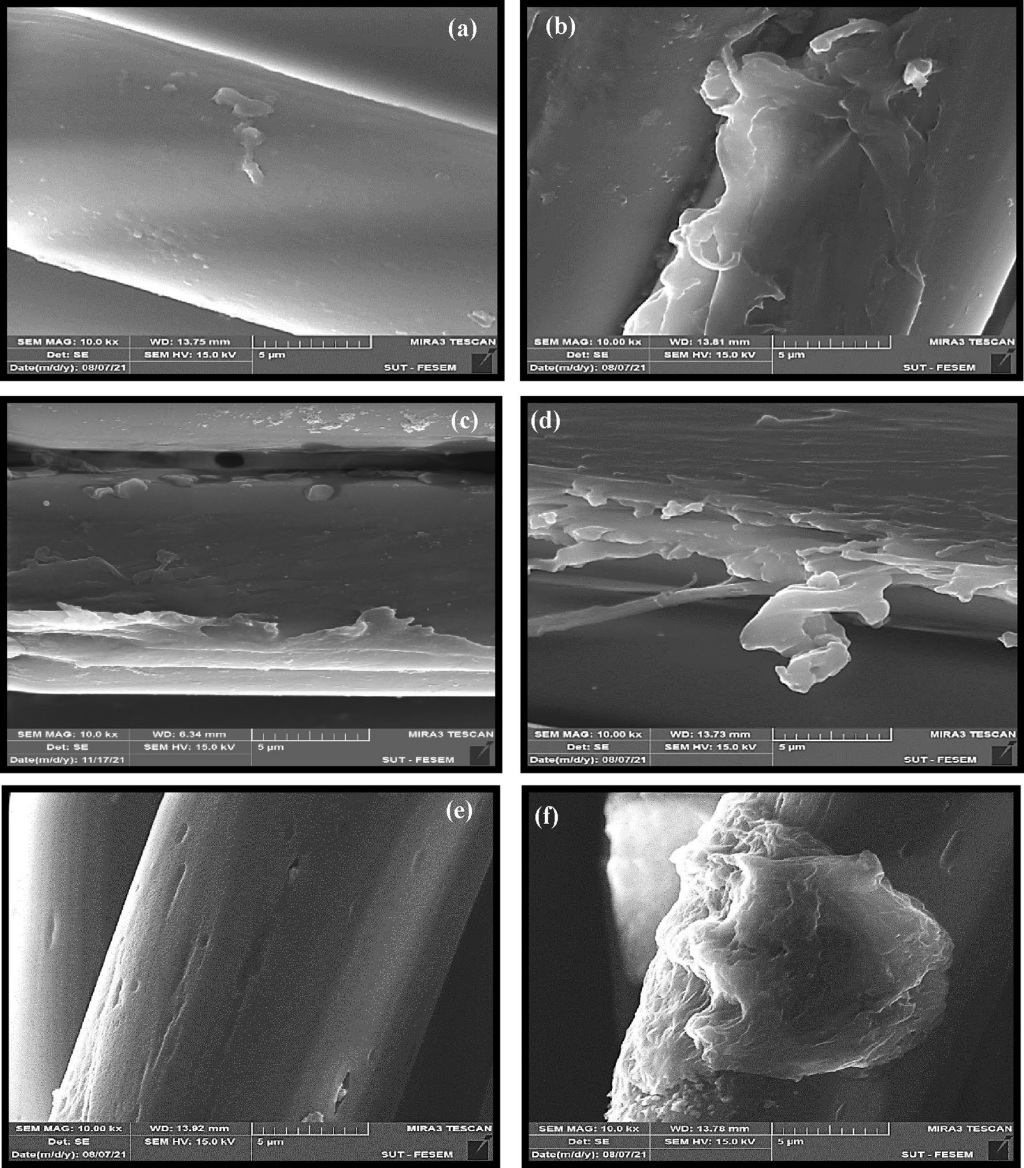
FE-SEM micrographs of PET fabrics; (**a**) pristine PET, (**b**) PET-GAP60, (**c**) PET-GAP90, (**d**) PET-GAP120, (**e**) PET-NaOH60 and (**f**) PET-NaOH120 samples.

SEM micrographs of carboxylated and hydroxylated PET fabrics were investigated. Figure 4a illustrates the smooth surface of pristine PET fiber. Figure 4b,d depict that photochemical functionalization caused destructions on the fiber surface. Based on the SEM images and the tensile results, it could be assumed that the UV irradiation caused degradation of just surface layer of the PET fibers. On this basis, these degradations could be considered as non-uniformities that improve the mechanical interlocking (i.e. bonding) between fibers and rubber matrix. It is obviously seen that hydrolysis by alkali solution caused surface grooves and severe destructions, especially in the sample that was exposed for 120 min to NaOH (see Fig. 4e,f).

Table 4 shows the elemental characteristics (i.e. EDS results) of the functionalized fabrics. Results confirmed the functionalization of PET by both carboxylation and hydroxylation methods due to the increment in the elemental oxygen.

**Table 4:** The elemental analysis data of the functionalized PET fabrics.

Sample	Element			
	C (± σ)	O (± σ)	Na (± σ)	Si (± σ)
Pristine PET	74.23 (± 0.19)	25.77 (± 0.19)	–	–
PET-GAP-90	71.3 (± 0.2)	27.7 (± 0.2)	–	–
PET-GAP-90-S	76.07 (± 0.2)	23.85 (± 0.2)	–	0.08 (± 0.02)
PET-NaOH60	70.1 (± 0.2)	28.39 (± 0.2)	1.51 (± 0.03)	–
PET-NaOH60-S	73.24 (± 0.19)	26.72 (± 0.19)	–	0.04 (± 0.02)

### Effect of silanization of PET fabrics

Based on the results of analysis and tensile properties, PET-GAP90 and PET-NaOH60 samples were selected as the best functionalized fabrics for the silanization step.

**Figure 5:**
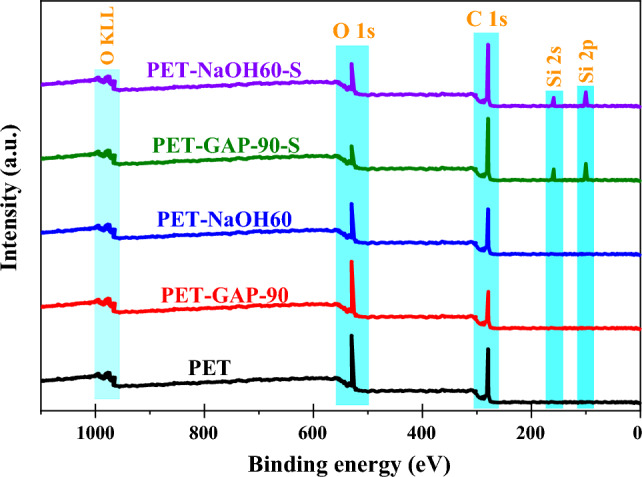
XPS spectra of PET fabric before and after treatments for Pristine PET, PET-NaOH60, PET-NaOH60-S, PET-GAP-90 and PET-GAP-90-S.

XPS analysis of the pristine, hydroxylated, carboxylated and VTMS-modified PET fabric is shown in Fig. 5. The results show the intact PET fabric with two peaks of C1s and O1s in the PET graph. In the PET-GAP-90 sample, the amounts of C1s and O1s decreased and increased, respectively. Also, in the PET-NaOH60 sample, the amounts of C1s and O1s decreased. After surface modification with silane, two new peaks (i.e. Si2p and Si2s) were formed in both samples, which confirmed the successful surface modification of the PET fabric. In both samples modified with silane (i.e. PET-GAP-90-S and PET-NaOH60-S), the amount of C1s decreased greatly, which indicates the formation of Si–O–Si and Si–OH bonds.

**Figure 6:**
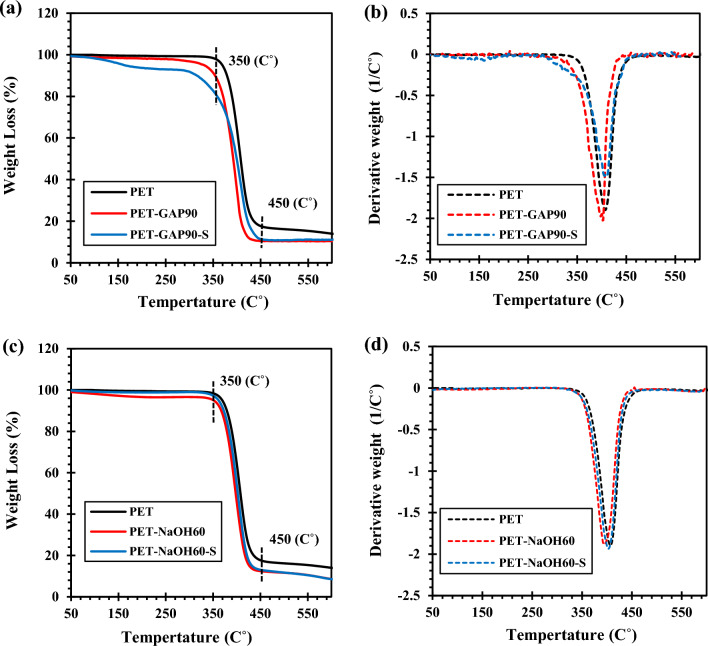
TGA and DTG results of the silanized PET fabrics functionalized by; (**a**,**b**) GAP/UV irradiation and (**c**,**d**) NaOH.

Figure 6 and Table 5 present TGA results for the silanized samples (i.e. PET-GAP90-S and PET-NaOH60-S). Results show that the silanization of the GAP/UV irradiated PET fabrics caused an increment in the weight loss of the sample at the temperature range of 120–350 (see Fig. 6a). This weight loss was attributed to degradation of grafted VTMS to the PET surface. It is obviously seen that the grafting of silane caused an increment in the thermal stability and the degradation temperature of the functionalized fibers (i.e. at temperature range of 350–450 °C) (see Fig. 6b). It was attributed to formation of an inorganic–organic layer on the PET surface.

**Table 5:** TGA Data for the silanized samples.

Sample code	Weight loss (%) at temperature range of			Grafting ratio
	120–350 (°C)	350–450 (°C)	450–600 (°C)	(%)
PET	0.24	84.4	1.35	–
PET-GAP90	7.35	80.73	0.1	–
PET-GAP90-S	14.88	71.2	0.33	9.53
PET-NaOH60	2.6	82.73	3.68	–
PET-NaOH60-S	1.75	84.23	4.48	1.5

The silanization also improved thermal stability of the NaOH functionalized PET fabric (see Fig. 6c,d). However, the silane grafting on hydroxylated sample was so much lower than GAP/UV irradiated PET (see Table 5). This was attributed to lower OH groups that created on the PET fiber during alkali treatment and this decreased chance of VTMS grafting. The silane grafting ratios of the silanized PET fabrics are shown in Table 5.

**Figure 7:**
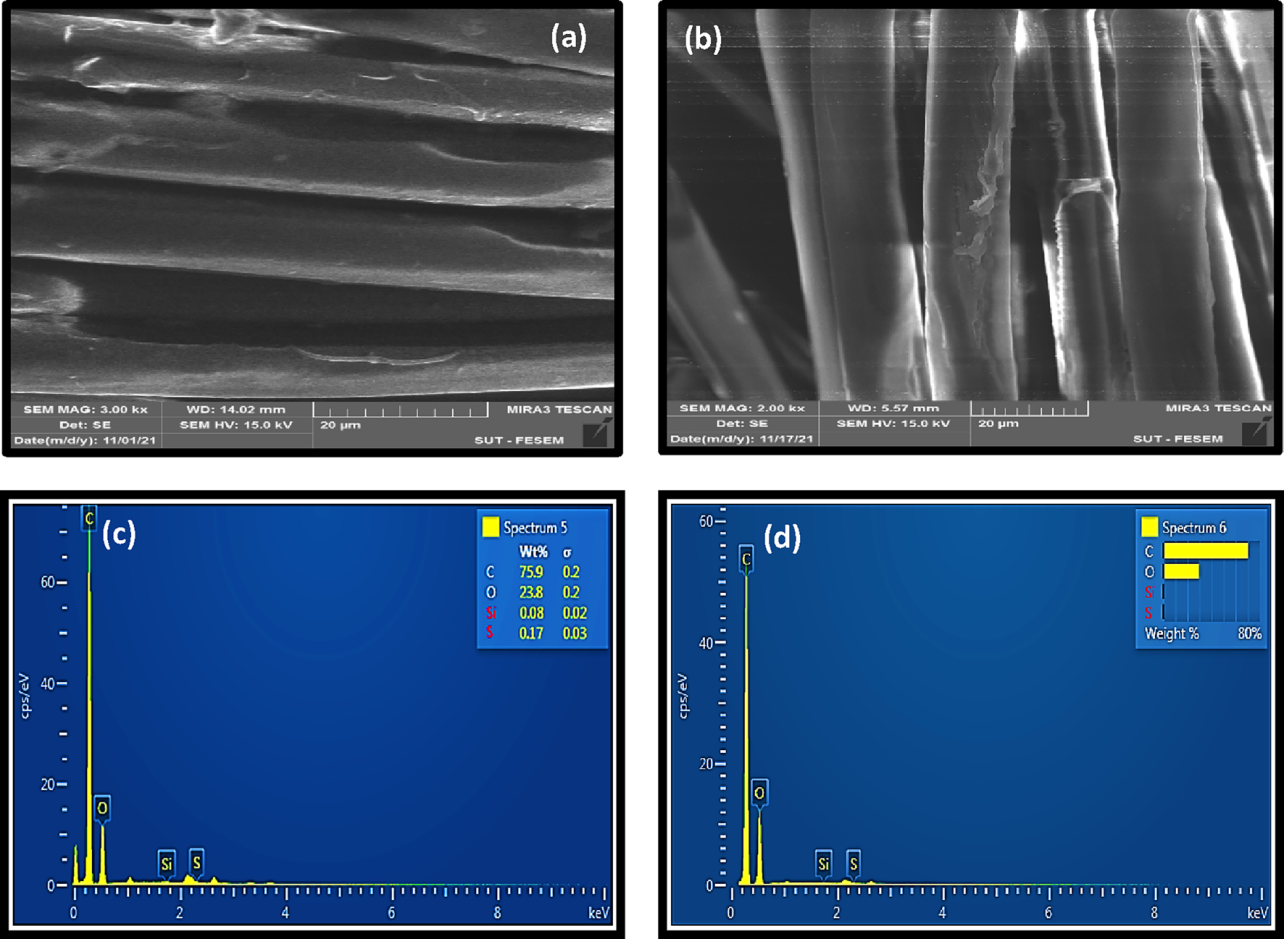
(**a**,**b**) FE-SEM images and (**c**,**d**) EDS results for the silanized samples with different functionalized PET fabrics.

Figure 7 illustrates FE-SEM images and EDS analysis of the silanized fabrics. The images obviously showed that the surface of both samples was covered by a condensed silane layer that caused the disappearance of non-uniformities (see Fig. 7a,b).

The elemental analysis (see Fig. 7c,d and Table 4) also confirmed successful grafting of the silane on the functionalized PET surfaces due to showing the presence of Si and C elements (i.e. these elements exist in the VTMS structure). However, the content of the element was very low in the case of alkali treatment.

### Adhesion tests

#### H-Pull test results

**Figure 8:**
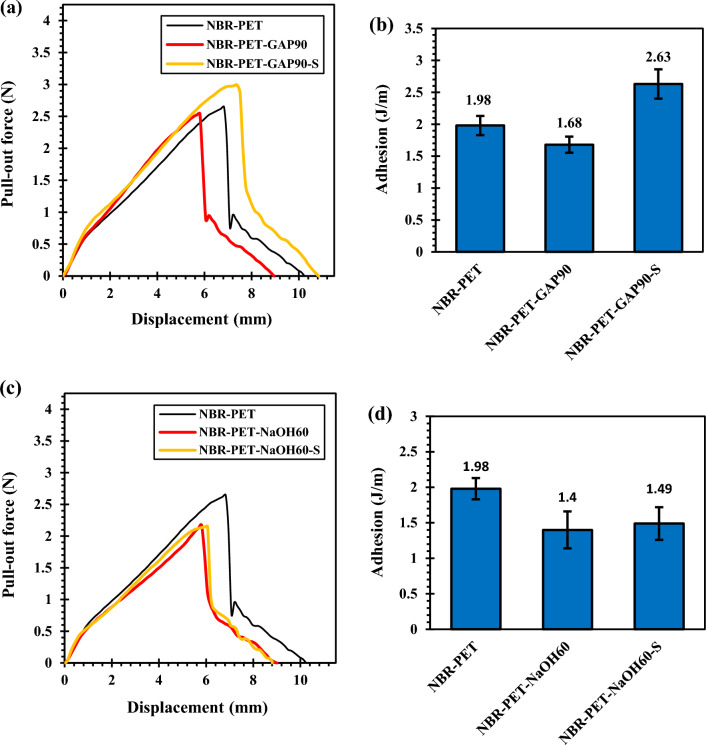
Pull out behavior of the PET fibers from NBR matrix; (**a**,**b**) functionalized by GAP/UV irradiation and (**c**,**d**) functionalized by NaOH.

Figure 8 shows the pullout behavior of functionalized and silanized PET cords from NBR matrix. The pullout adhesion of the samples were calculated from pullout load–displacement curves (i.e. area under the curves). Figure 8a,b clearly demonstrate that by functionalization of PET surface by GAP/UV irradiation, the pullout force and adhesion of fibers to NBR decreased. This corresponded to the creation of polar groups (i.e. COOH) on the PET surface by functionalization process that decreased its interfacial interactions with the nonpolar rubber matrix.

However, the GAP/UV irradiated PET cords showed improved adhesion after silanization (see Fig. 8b). This was attributed to contribution of vinyl groups of silane (i.e. VTMS) in vulcanization process of NBR that bonded PET chemically to the rubber matrix^44,45^.

Figures 8c,d depicts the pullout behavior of hydroxylated and silanized PET cords from NBR. It is seen that hydroxylation caused a decrement in the pullout force and adhesion. This was attributed to the creation of OH groups on the PET surface that made it hydrophilic and declined bonding to rubber matrix. In contrast to GAP/UV irradiated PET, silanization of hydroxylated PET did not cause an increment in the adhesion. This was attributed to the very low silane grafting content and also severe destructions occurred in this sample during alkali treatment that decreased the fibers strength. In fact, these fibers were ruptured during the pullout test.

**Figure 9:**
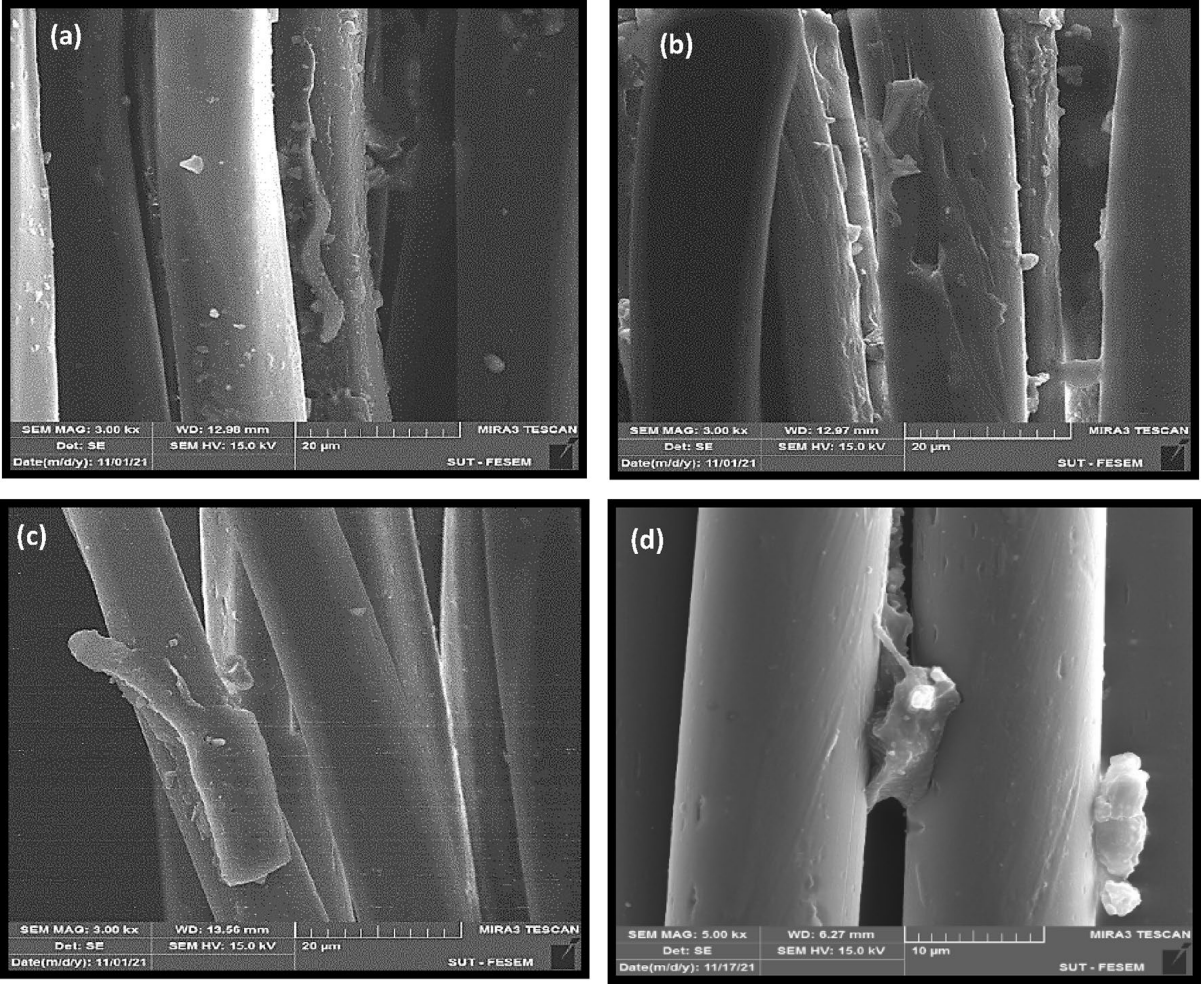
SEM images from the surface of the pulled out cords; (**a**) PET-GAP90, (**b**) PET-GAP90-S, (**c**) PET-NaOH60 and (**d**) PET-NaOH60-S.

SEM images show the surface of pulled-out cords. It is clearly seen that there are a little rubber particles on the surface of the functionalized fibers before silanization (see Fig. 9a,c). After silanization, the rubber adhered to the fibers surface and diffused between them especially in the GAP/UV irradiated sample (see Fig. 9b). Figure 9d shows that the rubber diffused between the alkali-treated fibers but did not cover the fibers surface. This confirmed the results of the pull-out adhesion test.

#### T-peel test results

**Figure 10:**
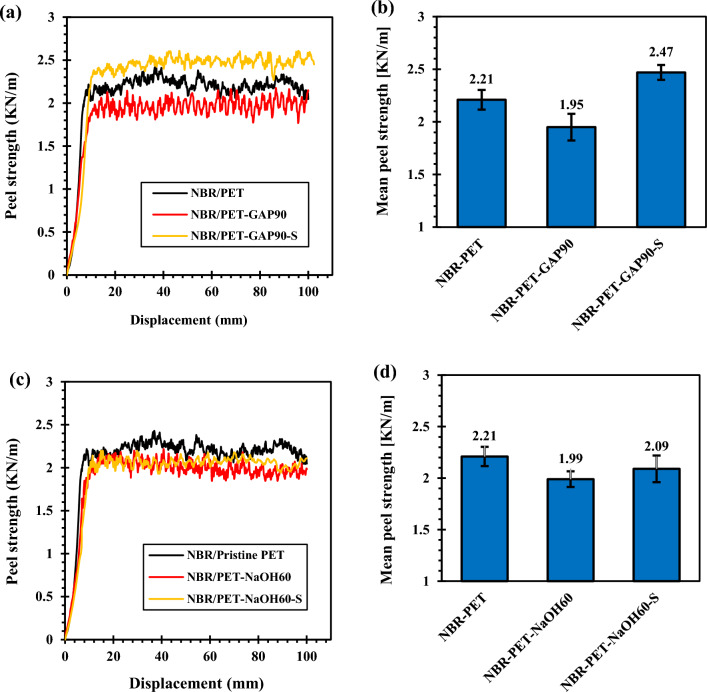
Peel test results for the PET fibers with different functionalization; (**a**,**b**) GAP/UV irradiation and (**c**,**d**) NaOH.

Figure 10 shows the T-peel test results. It is clearly seen that functionalizations had negative effects on the T-peel adhesion of PET fabrics to NBR that was in good correlation with H-pull test results. However, GAP/UV irradiated PET fabric showed an increment in the T-peel adhesion after silanization while silanization had no influence on the adhesion of alkali-treated PET to NBR. This was attributed to the damages that occurred in alkali-treated PET fibers that caused their rupture during the T-peel test (i.e. cohesion defect in PET fibers instead of interfacial fracture).

**Figure 11:**
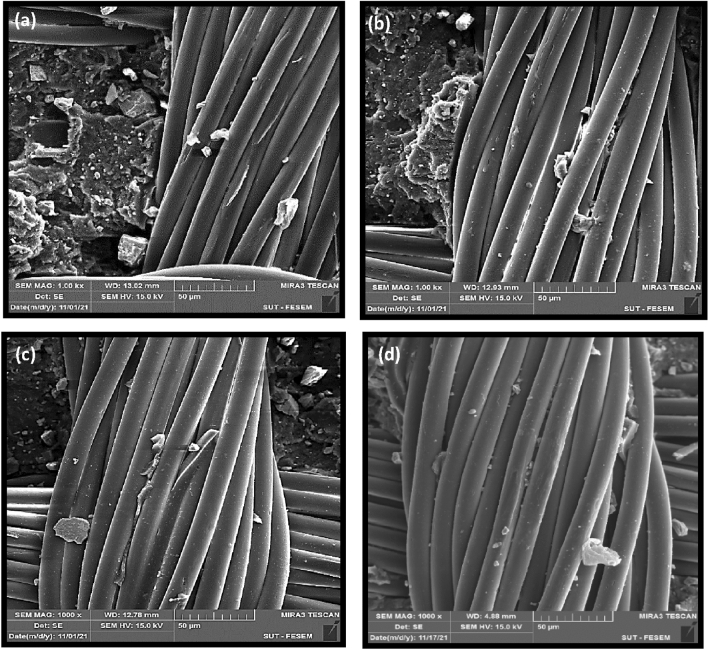
SEM images from the surface of the T-peeled fabrics; (**a**) PET-GAP90, (**b**) PET-GAP90-S, (**c**) PET-NaOH60 and (**d**) PET-NaOH60-S.

**Figure 12:**
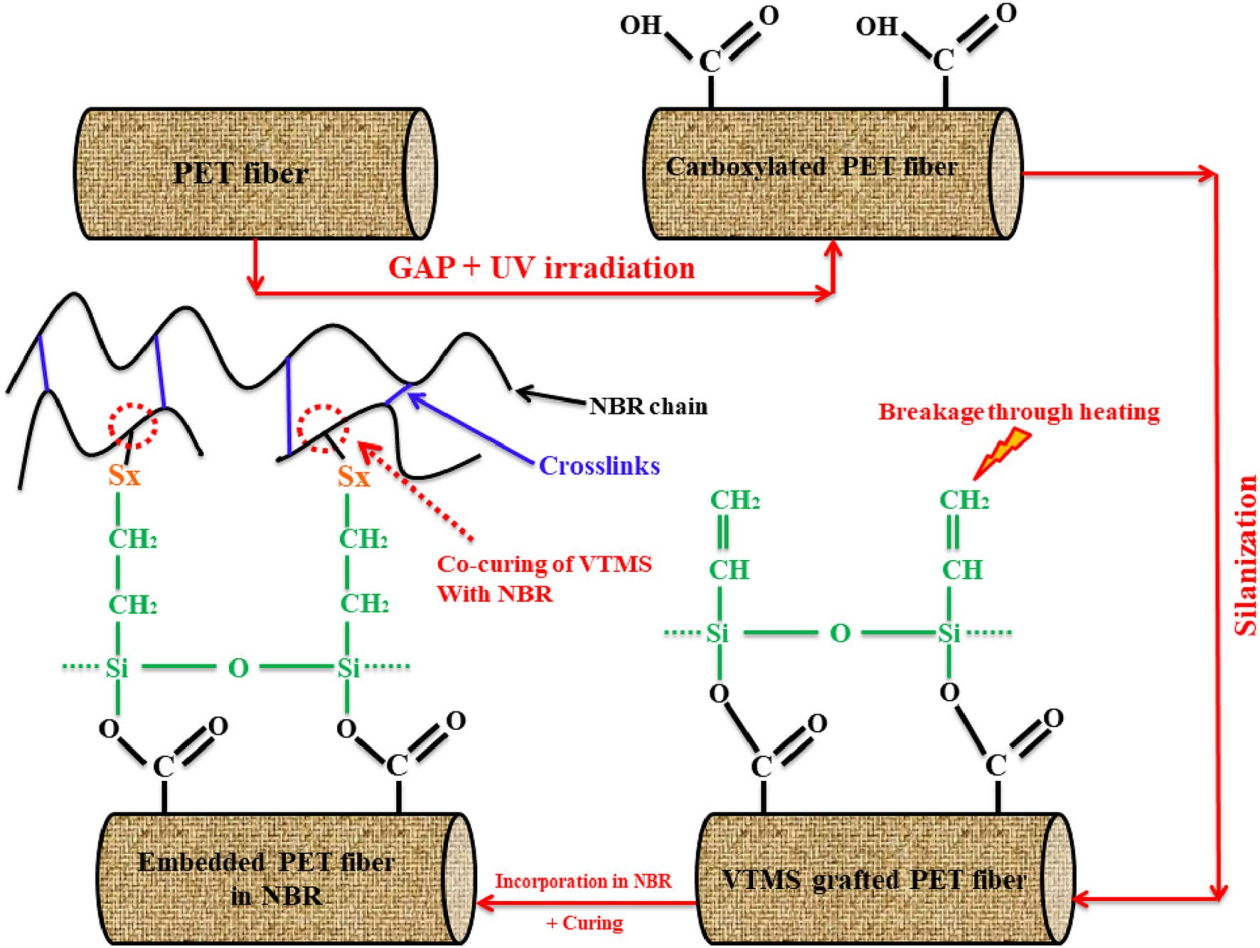
Schematic for surface functionalization of PET using GAP/UV irradiation and interfacial interactions between surface modified PET and NBR interface.

Figure 11 discloses SEM images of the PET fabrics after T-peel adhesion. The images show that rubber diffused between the filaments and appeared more on the surface after silanization. Figure 12 illustrates a schematic for interfacial interactions between silanized GAP/UV irradiated PET surface and NBR.

## Conclusions

In this work, the effect of chemical (i.e. by NaOH solution) and photochemical (i.e. by GAP/UV irradiation) functionalization on the thermo-mechanical properties of PET fabric and its adhesion to nitrile rubber (NBR) was compared. By increasing the hydrolysis time in NaOH solution from 60 to 120 min, the OH content decreased 5 times and the tensile strength and modulus decreased by 11 and 9%, respectively. Also, increasing the exposure time of PET fabric to UV irradiation by more than 90 min caused a reduction in the OH content by 29% and increased the tensile strength by 3.5%. Photochemical functionalization for 90 min created much more OH groups on the PET fabric surface (i.e. 4.5 times) compared to the hydrolysis of PET fabric in NaOH for 60 min. It also increased the tensile strength of PET fabric by 5% compared to pristine PET while the hydroxylated PET fabric showed a slight decrement in the tensile strength. The results showed the functionalization of PET fabric by GAP under UV irradiation caused an increment in the silane grafting ratio up to 6.5 times compared to the hydrolyzed fabric. In the following, it was found that chemical and photochemical functionalization methods caused decrement in the pullout adhesion by about 30 and 15%, respectively. They also caused about 10% decrement in the T-peel adhesions to nitrile rubber. Functionalization of PET fabric by GAP/UV irradiation and then silane grafting caused 33 and 12% increments in the pullout and T-peel adhesion of PET to NBR, respectively. However, silanization had not the same impact on the hydrolyzed fabrics due to the lower silane grafting content and also more damages occurred during the functionalization process.

## Data Availability

It is confirmed that all Data Availability. The raw/processed data required to reproduce these findings can be shared. To request data, contact Dr. Jamshidi by email: Masoud52@yahoo.com.
